# Factors Associated With Time to Achieve Employment Through Occupational Support Programs in Patients With Mood Disorders: 1 Year Naturalistic Study

**DOI:** 10.3389/fpsyt.2021.617640

**Published:** 2021-03-16

**Authors:** Tomonari Hayasaka, Yoshikazu Takaesu, Izumi Nagashima, Miku Futada, Kazuhiro Nozaki, Takeshi Katagiri, Yayoi Imamura, Mariko Kurihara, Yuki Oe, Takashi Tsuboi, Koichiro Watanabe

**Affiliations:** ^1^Department of Occupational Therapy, Faculty of Health Sciences, Kyorin University, Tokyo, Japan; ^2^Department of Neuropsychiatry, School of Medicine, Kyorin University, Tokyo, Japan; ^3^Department of Neuropsychiatry, Kyorin University Hospital, Tokyo, Japan; ^4^Department of Neuropsychiatry, Graduate School of Medicine, University of Ryukyus, Okinawa, Japan

**Keywords:** mood disorders, major depressive disorder, bipolar disorder, occupational therapy, occupational support program

## Abstract

**Objective:** Mood disorders cause significant work performance disability in sufferers and often lead to adverse employment outcomes in working individuals. The aim of this study was to explore factors associated with time to achieve employment through the occupational support program (OSP) for patients with mood disorders.

**Methods:** The participants were patients admitted to the Kyorin university hospital from April 2016 to April 2019. Patients who met the criteria for major depressive disorder and depressive episode of bipolar I or II disorder according to DSM-5 and participated in the occupational therapy-based OSP for at least three sessions (one course) were included in this study. We collected demographic and clinical variables at the baseline of this study through medical records and OSP records; the variables included age, gender, diagnosis, scores of Quick Inventory of Depressive Symptomatology and Global Assessment of Functioning, the number of times of participation in the OSP, word count of the transcription task in the OSP, typographical deficiency, fatigue status and mood status after the OSP. The primary outcome was set as the time to achieve the employment within 1 year after the discharge.

**Results:** Of the 211 patients who participated in the OSP during the survey period, 49 participants met the criteria in this study. The results showed that 14 patients achieved and the other 35 patients did not achieve the employment within 1 year of discharge from the hospital. A multivariate cox regression analysis revealed that the word count of the transcription task in the OSP (HR = 1.03, 95% CI = 1.01–1.05, *p* = 0.016) and mood status after the OSP (HR = 2.77, 95% CI = 1.18–6.51, *p* = 0.019) were significantly associated with time to achieve the employment.

**Conclusion:** In conclusion, this study suggested that work speed and mood response in the OSP could be significant predictors for achieving employment in patients with mood disorders.

## Introduction

Mood disorders are one of the most detrimental mental diseases to employment, and the reduced productivity due to sick leave or retirement is a major burden for companies (1–3). The Global Burden of Disease Study has shown that mood disorders cause economic losses to the society (4). Mood disorders are a highly recurrent mental disease (5–8), with a 12-month prevalence rate of 3.4–6.0% in the working population in European countries (9, 10), and 6.4% in the same population in the United States (11), indicating that many individuals and companies are faced with problems caused by mood disorders.

While there are increasing expectations for specific measures to address these problems (12), companies and healthcare institutions have come together in recent years to improve the employment support system (13, 14). In particular, the effectiveness of rehabilitation programs for the employment support has been verified by several survey studies and interventional studies (15, 16). On the other hand, specific methods of employment support for patients with mood disorders and its effectiveness have not been fully verified, and there is a call for the establishment of employment support strategies for patients with mood disorders.

Previous studies have reported several factors associated with lower employment rates in people with mood disorders. Comorbid physical illnesses (17) and prolonged disease duration (18) seen in these patients suggest that the severity of the disorder or disability is associated with employment, and the frequency of past sick leave and unemployment (19) are associated rather with the continuation of work after employment than with the employment rate. As for demographic variables such as sex, academic background (20), and age (21), their associations with the employment rate are thought to vary according to the type of work. The association between these factors and employment has not been thoroughly examined to date, and the lack of predictive factors associated with employment has been pointed out (22). To predict whether the patient will be able to work during the clinical course of mood disorders, work performance indices for employment are necessary. Only a few studies have focused on work performance in patients with mood disorders and examined its association with employment.

Accordingly, this study examined patients with mood disorders who were enrolled in an inpatient employment support program and explored factors associated with time to achieve employment using a 1-year post-discharge follow-up survey.

## Materials and Methods

### Participants

This study was approved by the ethics committee of the School of Medicine, Kyorin University (approval number: R02-109). The participants were patients admitted to the department of psychiatry in Kyorin University Hospital from April 2016 to March 2019. Inclusion criteria for this study were as follows; (a) patients who were not working at the time of admission (including unemployment and sick leave) and were seeking employment, (b) patients who met the criteria for major depressive disorder and depressive episode of bipolar I or II disorder according to DSM-5, (c) patients who participated in the occupational therapy-based occupational support program (OSP) for at least three sessions (one course), (d) patients between 18 and 60 years old. Exclusion criteria included the following: patients who had (a) ongoing alcohol or substance abuse, (b) suicidal risk, (c) schizophrenia, (d) dementia, (e) severe physical disease.

### Outline of the OSP

The primary purpose of OSP was to assess cognitive function and work performance of participants. The OSP was conducted every Tuesday by one occupational therapist and was a group session with ~6 inpatients participating. The main content was the task of transcription a newspaper article published on the day. The task was set at a time limit of 5 min. Then, we checked the number of characters transcribed and typographical errors among participants. In the group session, participants were asked to answer a questionnaire which had questions about their fatigue and mood status with five-point scale responses. Table 1 shows the details of the OSP.

**Table 1:** Details of the implementation of the occupational support program.

**Procedure of the OSP**	**Program details**
(a) Start of the program	Promotion of motivation for participation and confirmation of participation
(b) Description of the program	Confirmation of content and purpose of the session
(c) Icebreaker	Perform simple figure puzzles and games
(d) Implementation of the transcription task	Transcription of a newspaper article published on the day of implementation on manuscript paper Participants were asked to answer and self-evaluate their fatigue status after the transcription task
(e) End of the program	Present your goals for the next session. Participants were asked to answer and self-evaluate their mood status after the session

*OSP, Occupational Support Program. Occupational support program has three sessions (3 weeks) per course. Occupational support program was performed in procedure (a)–(e)*.

### Study Design and Survey Contents

The study was conducted as a retrospective survey. We collected demographic and clinical variables at the baseline of this study through medical records and OSP records; the variables included age, gender, diagnosis of psychiatric disorder, scores of Quick Inventory of Depressive Symptomatology (QIDS-SR). There are 16 items in the QIDS-SR measuring the nine criterion symptom domains (sleep, mood, weight, concentration, guilt, suicidal Ideation, interest, fatigue, and psychomotor Changes) that define a major depressive episode according to the DSM-IV. Additionally, Global Assessment of Functioning (GAF), number of times of participation in the OSP, word count in the 5-min transcription task (transcription task) at the first participation in the OSP, typographical deficiency, fatigue status after the transcription task, and mood status after the first participation in the OSP were recorded. The scores of fatigue status ranged from one (no difficulty) to five (severe difficulty) and mood status ranged from one (very bad) to five (very good).

The primary outcome was set as the time to achieve employment within 1 year of discharge. “Achieve employment” in the study was defined as full-time employment, part-time employment, casual employment, and sheltered work. We divided the participants into two groups, namely, participants who met the criteria of achieving employment during the 1 year after the discharge (employment group), and participants who did not meet the criteria as above (non-employment group).

### Statistical Analysis

The Mann-Whitney *U*-test and chi-square test or Fisher's exact test were used for the comparison of the employment group and non-employment group for age, gender, diagnosis, QIDS-SR, GAF, number of times of participation in the OSP, word count in the transcription task of the first participation in the OSP, typographical deficiency, fatigue status after the transcription task, and mood status after the first participation in the OSP between the employment group and non-employment group. For the analysis focused on the achieving employment, Kaplan-Meier survival curves were used to compare the time to achieving employment among the participants.

Pearson's correlation coefficient was used to examine the association between each criterion symptom of QIDS-SR and the OSP scores.

A multivariate Cox proportional hazard model was used to examine the association between individual predictors and time to achieve employment. We selected independent variables associated with the OSP. The following variables were entered as independent variables: number of times of participation in the OSP, word count in the transcription task of the first participation to the OSP, typographical deficiency, fatigue status after the transcription task and mood status after the first participation to the OSP. SPSS version 23 software for Windows (SPSS Inc., Chicago) was used for all statistical analyses. A *p*-value of < 0.05 was considered to indicate a statistically significant difference.

## Results

**Figure 1:**
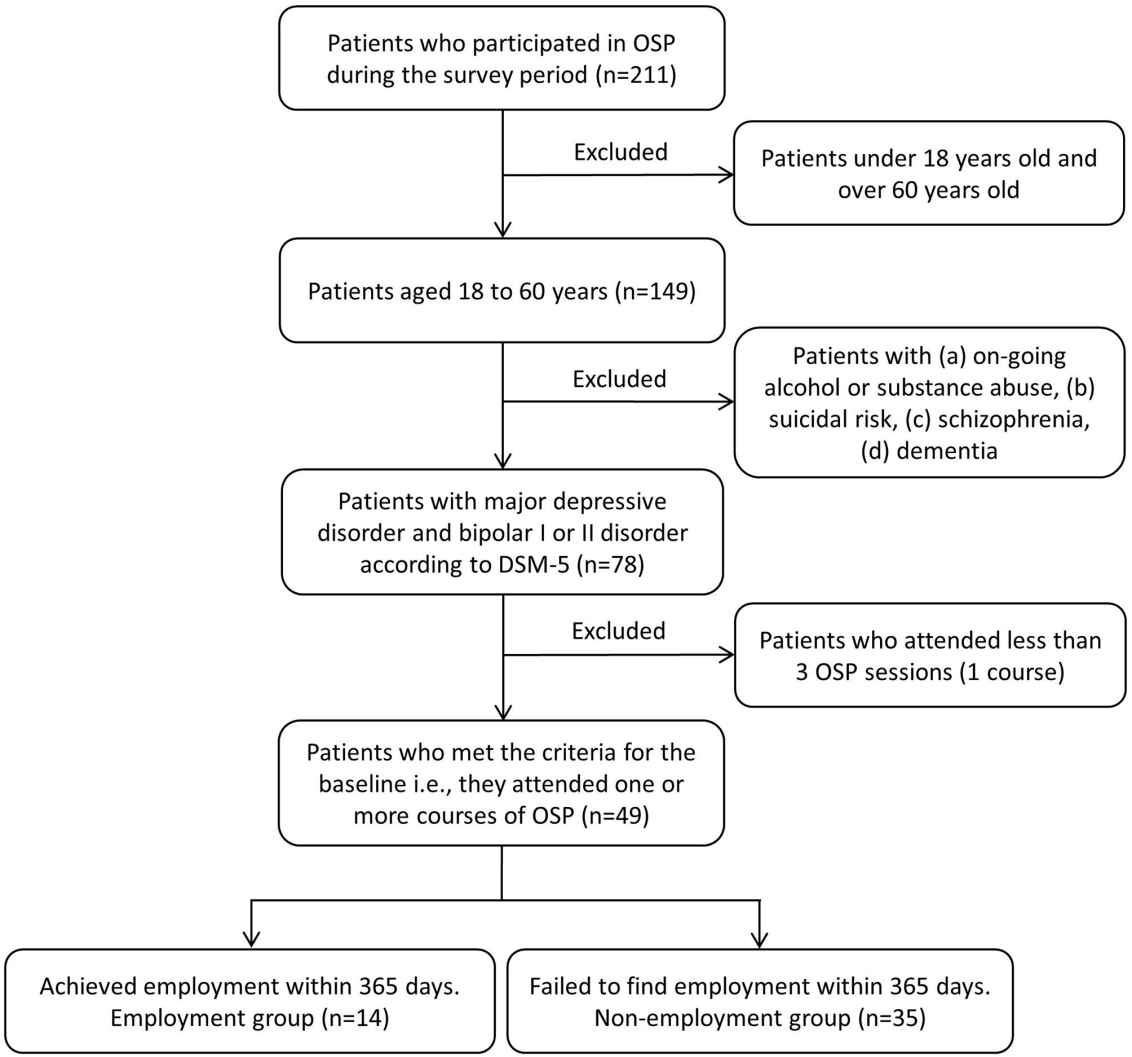
Flowchart for the exclusion procedure. OSP, Occupational Support Program.

Of the 211 patients who participated in the OSP during the survey period, 49 participants (male = 17, female = 32, age = 44.89 ± 10.49) met the criteria in this study at the baseline. Figure 1 shows the flow chart of the study participants based on the inclusion and exclusion criteria.

Fourteen patients achieved employment (employment group) and 35 patients did not achieve employment (non-employment group) within a year of discharge from the hospital. Twenty-eight patients were diagnosed with major depressive disorder (MDD) and 21 patients were diagnosed with bipolar disorder (BP). Among the comparisons of demographics and clinical variables for the baseline in the study between the employment group and non-employment group, the word count in the transcription task at the first participation in the OSP (131.79 ± 23.73 vs. 106.20 ± 39.23) and mood status after the first of the first participation to the OSP (3.64 ± 0.63 vs. 3.09 ± 1.07, *p* = 0.03) in the employment group were significantly higher than those in the non-employment group (Table 2). No significant difference in other variables was found between the two groups (Table 2). There was a significant negative correlation between the weight subscore of QIDS-SR and the word count of OSP. Additionally, there was a significant negative correlation between fatigue subscore of QIDS-SR and the number of times of participation of OSP (Table 3). However, there was a significant positive correlation between mood status and the number of times of participation in the OSP (Table 4).

**Table 2:** Comparison of demographic and clinical valuables of the participants between the employment group and non-employment group.

**Variables**	**Mean and standard deviation and each ratio of each group**		***P*-value**
	**Employment group** **(*n* = 14)**	**Non-employment group** **(*n* = 35)**	
Age at the time of first discharge (years)	43.43 ± 10.41	46.34 ± 10.56	0.387
Sex (male: female)	4:10	13:22	0.743
Diagnosis (MDD: BD)	6:8	22:13	0.222
QIDS-SR scores at the time of hospitalization (points)	15.23 ± 5.82	16.75 ± 3.98	0.400
GAF scores at the time of hospitalization (points)	28.79 ± 8.35	28.54 ± 4.81	0.920
Number of times of participation in the OSP (times)	5.36 ± 1.82	4.94 ± 2.11	0.499
Mood status after the first participation in the OSP (points)	3.64 ± 0.63	3.09 ± 1.07	0.030^*^
Word count in the transcription task at the first participation in the OSP (words)	131.79 ± 23.73	106.20 ± 39.23	0.008^**^
Number of typographical errors in the transcription task at the first participation in the OSP (words)	1.07 ± 1.14	1.31 ± 3.31	0.705
Fatigue status after the first participation in the transcription task (points)	2.86 ± 0.77	2.76 ± 1.24	0.735

*MDD, Major Depressive Disorder; BD, Bipolar Disorder; QIDS-SR, Quick Inventory of Depressive Symptomatology (Self-Report); GAF, Global Assessment of Functioning; OT, Occupational Therapy; OSP, Occupational Support Program*.

*The value represents the mean ± SD. Mann-Whitney U-test was used to compare the continuous variables between the two groups. The chi-square test was used to compare categorical variables*.

* *p < 0.05*,

** *p < 0.01*.

**Table 3:** Pearson's correlation coefficient between QIDS-SR and OSP scores.

	**OSP scores**				
	**Number of participation**	**Mood status**	**Word count**	**Typographical errors**	**Fatigue status**
QIDS-SR total	−0.04	−0.07	−0.12	−0.09	0.01
Sleep	0.01	−0.18	0.25	0.02	−0.09
Mood	0.06	0.18	−0.18	−0.19	0.02
Weight	−0.15	−0.01	−0.45^**^	0.02	−0.11
Concentration	0.02	0.01	−0.17	−0.11	0.15
Guilt	0.14	−0.04	0.06	0.10	0.12
Suicidal ideation	0.04	0.05	−0.02	−0.09	−0.09
Interest	−0.26	−0.15	−0.14	−0.14	0.03
Fatigue	−0.31^*^	−0.19	0.12	0.08	0.03
Psychomotor slowing	0.08	−0.12	0.01	−0.15	−0.01

p = Pearson's correlation coefficient

* *p < 0.05*,

** *p < 0.01. QIDS-SR, Quick Inventory of Depressive Symptomatology (Self-Report) scores at the time of hospitalization. Number of participation: Number of times of participation in the OSP. Mood status: Mood status after the first participation in the OSP. Word count: Word count in the transcription task at the first participation in the OSP. Typographical errors: Number of typographical errors in the transcription task at the first participation in the OSP. Fatigue status: Fatigue status after the first participation in the transcription task. OSP, Occupational Support Program*.

**Table 4:** Pearson's correlation coefficient between each OSP scores.

	**Number of participation**	**Mood status**	**Word count**	**Typographical errors**	**Fatigue status**
Number of participation	1.00	0.32^*^	0.05	−0.17	0.13
Mood status		1.00	−0.14	−0.27	−0.15
Word count			1.00	0.24	−0.23
Typographical errors				1.00	−0.09
Fatigue status					1.00

p = Pearson's correlation coefficient

* *p < 0.05. Number of participation: Number of times of participation in the OSP. Mood status: Mood status after the first participation in the OSP. Word count: Word count in the transcription task at the first participation in the OSP. Typographical errors: Number of typographical errors in the transcription task at the first participation in the OSP. Fatigue status: Fatigue status after the first participation in the transcription task. OSP, Occupational Support Program*.

Of the 49 participants, 14 participants achieved employment within 1 year of discharge. The average number of days before achieving employment in the employment group by the Kaplan-Meier survival estimate was 156.1 ± 108.4 days.

Survival analysis was also performed using Cox proportional hazards regression in a multivariate model. We selected the following independent variables at the baseline (number of times of participation in the OSP, word count in the transcription task of the first participation in the OSP, typographical deficiency, fatigue status after the transcription task and mood status after the first participation in the OSP). As show in Table 2, the word count in the transcription task of the first participation in the OSP (HR = 1.03, 95% CI = 1.01–1.05, *p* = 0.016) and mood status after the first participation in the OSP (HR = 2.77, 95% CI = 1.18–6.51, *p* = 0.019) were significantly associated with the time to achieve employment of the participants (Table 5).

**Table 5:** Multivariate Cox hazard proportional analysis for factors associated with time to achieve employment.

**Analytic factors**	**Hazard ratio (95% CI)**	***P*-value**
Number of times of participation in the OSP	0.88 (0.67–1.15)	0.340
Mood status after the first participation in the OSP	2.77 (1.18–6.51)	0.019^*^
Word count in the transcription task at the first participation in the OSP	1.03 (1.01–1.05)	0.016^*^
Number of typographical errors in the transcription task at the first participation in the OSP	0.89 (0.52–1.53)	0.677
Fatigue status after the first participation in the transcription task	0.85 (0.36–2.00)	0.709

*OSP, Occupational Support Program. 95%CI = 95% confidence interval*.

* *p < 0.05*.

## Discussion

To the best of our knowledge, this is the first study ever to examine factors associated with the time to achieve employment in people with mood disorders who are enrolled in an employment support program. This study examines what type of work performance skills of inpatients with mood disorders can predict their post-discharge employment status. The results showed that the word count in the transcription task and mood after the transcription task were predictive of achieving employment within 1 year of discharge.

Previous studies have reported that the severity of depressive symptoms and prolonged disease duration in depressed patients affect their employment (18). Depressed workers and non-workers differ not only in demographic variables such as age, academic background (20, 21), but also in the perception of their own health status, with depressed workers perceiving the inconvenience caused by decreased physical activities less often than depressed non-workers (23). Although participants in this study presented with moderate to severe depressive symptoms and systemic dysfunctions, there were no significant differences in QIDS-SR and GAF between the employment and non-employment groups. A previous study reported that the alleviation of symptoms only partially reduced adverse effects on employment (19), suggesting that these scales may not necessarily be predictive of post-discharge employment status.

The productivity losses that depression causes to society have been shown in a comparative study with healthy workers which reported that factors associated with performance skills at work account for 80% of these losses (23). Likewise, a study of presenteeism among depressed workers found that the severity of depression affected the decline in work performance and work performance was associated with employment limitations. These findings suggest that the comprehensive assessment of work performance is essential in examining individual and economic burdens (24, 25). Another study on work performance and employment limitations in depressed patients found significant impairments in the ability to perform tasks that involve psychological and interpersonal relationships, and the ability to manage work schedules and finish tasks on time (26). Similar to our study results, previous studies have reported the association between work performance and employment, but not the association between work speed and employment status. The transcription task of the present study measures the speed to transcription as many characters as possible. Since work speed is a vital work performance skill that generates good employment productivity, we can hypothesize that impaired work speed is associated with employment status and reduces productivity at work. Therefore, the assessment of work speed in inpatients with mood disorders can be used as a predictive factor for the possibility of post-discharge employment.

Meanwhile, an epidemiological study on absenteeism has reported that mood disorders are more likely to affect absenteeism than any other mental diseases (27). Among workers with mood disorders, the impaired ability to work is related to depression, and the prolonged disease duration significantly reduces work participation in middle- to older-aged workers (28). While some studies reported that relapse of mood disorders leads to the decline of absenteeism, a prospective study reported that remission of mood disorders allows continued full-time employment (29). For continued employment, work hour management and coordination of work schedules are essential. Studies on work hours and persistence of depressed mood have found an association between these factors. Overwork due to long working hours or working in hourly shifts allows depressive mood to persist and reduce adaptation to work (30). The present study found that patients with good mood reactivity after the OSP were able to work after discharge, suggesting that mood reactivity after the task can be used as an indicator to examine the adaptability to work.

This study has several limitations. First, since this study was conducted in a single university hospital with a small sample size, it is difficult to generalize these results to all patients with mood disorders. Second, since this was a retrospective study, the classification and notation of information have not been controlled, which may have generated bias in the quality of information. Third, our study conducted QIDS-SR and GAF on participants; however, the study participants' severity was not assessed in detail. Moreover, we did not evaluate mood status over time, before and after the OSP. Therefore, it prevented us from scrutinizing the differences in psychiatric symptoms between the working and non-working groups or mood status changes after OSP. Fourth, although each patient received different treatments than the occupational therapy, including drug therapy and psychotherapy, these factors were not assessed or examined for relevance. Fifth, the survey on employment status ran for a year after hospital discharge, it was impossible to run for a longer period due to consideration on the interventional period of the OSP. Sixth, in this study, we defined “achieve employment” as full-time, part-time, casual employment, and sheltered work. However, we did not survey the details of the employment statuses of participants. Therefore, further study will be needed to clarify the differences in “achieve employment.” Seventh, although the number of days of continuous employment after achieving employment is considered an essential issue in the field of occupational health, we did not evaluate this issue in this study. Specifically, Hori et al. suggested that social adaptation, working memory function, and benzodiazepine dosage are associated with the ability to continue working following a return to work after recovery from MDD (31). This issue should be considered in clinical settings when reviewing a patient's suitability for returning to work. Eighth, the employment rate in depressed outpatients who received support program within 1 year was reported to be ~70% in several studies (32–34). In contrast, the employment rate of the mood disorder in-patients who received OSP within 1 year was ~30% in this study. This discrepancy might be due to the severity of the mood disorder between our study and previous studies. Our study might have included more severe participants who were unemployed. Therefore, it was difficult to generalize this result to all patients with mood disorders. In light of these limitations, further examinations are needed to determine the association between work speed or mood reactivity and employment status, and factors relevant to time to achieving employment after discharge.

In conclusion, results of the present study suggested that the word count in the transcription task and mood status after the OSP can be factors associated with the employment status of patients with mood disorders for 1 year after discharge. The work speed affects the work performance, which further helps achieve employment, and mood reactivity after work was associated with the adaptability to work. For employment support for people with mood disorders, it is important to implement a rehabilitation program that is focused on work performance and workplace adaptability skills, in combination with the common treatment of depressive symptoms such as pharmacotherapy and/or psychotherapy. The results of the present study should be used for the promotion of employment among people with mood disorders and may contribute to the development of better employment support programs.

## Data Availability Statement

The original contributions presented in the study are included in the article/supplementary material, further inquiries can be directed to the corresponding author/s.

## Ethics Statement

The studies involving human participants were reviewed and approved by Faculty of Medicine Research Ethics Committee, Kyorin University. Written informed consent for participation was not required for this study in accordance with the national legislation and the institutional requirements.

## Author Contributions

TH, IN, YT, TT, and KW conceived the presented idea and designed the experiments. TH, MF, TK, YI, and KN carried out the experiments. TH, YT, MK, and YO analyzed the data. TH, YT, IN, MF, and KW interpreted the results. TH, YT, TT, and KW drafted the manuscript. All authors contributed to the article and approved the submitted version.

## Conflict of Interest

The authors declare that the research was conducted in the absence of any commercial or financial relationships that could be construed as a potential conflict of interest.
